# Role of Interleukin-6 in Vascular Health and Disease

**DOI:** 10.3389/fmolb.2021.641734

**Published:** 2021-03-16

**Authors:** Paulina Villar-Fincheira, Fernanda Sanhueza-Olivares, Ignacio Norambuena-Soto, Nicole Cancino-Arenas, Felipe Hernandez-Vargas, Rodrigo Troncoso, Luigi Gabrielli, Mario Chiong

**Affiliations:** ^1^ Advanced Center for Chronic Diseases & CEMC, Faculty of Chemical and Pharmaceutical Sciences, Universidad de Chile, Santiago, Chile; ^2^ Laboratorio de Investigación en Nutrición y Actividad Física (LABINAF), Instituto de Nutrición y Tecnología de los Alimentos, Universidad de Chile, Santiago, Chile; ^3^ Advanced Center for Chronic Diseases, Faculty of Medicine, Pontificia Universidad Católica de Chile, Santiago, Chile

**Keywords:** interleukin-6, soluble IL-6 receptor, gp130, exercise, vascular remodeling, vascular smooth muscle cells

## Abstract

IL-6 is usually described as a pleiotropic cytokine produced in response to tissue injury or infection. As a pro-inflammatory cytokine, IL-6 activates innate and adaptative immune responses. IL-6 is released in the innate immune response by leukocytes as well as stromal cells upon pattern recognition receptor activation. IL-6 then recruits immune cells and triggers B and T cell response. Dysregulated IL-6 activity is associated with pathologies involving chronic inflammation and autoimmunity, including atherosclerosis. However, IL-6 is also produced and released under beneficial conditions, such as exercise, where IL-6 is associated with the anti-inflammatory and metabolic effects coupled with physical adaptation to intense training. Exercise-associated IL-6 acts on adipose tissue to induce lipogenesis and on arteries to induce adaptative vascular remodeling. These divergent actions could be explained by complex signaling networks. Classical IL-6 signaling involves a membrane-bound IL-6 receptor and glycoprotein 130 (gp130), while trans-signaling relies on a soluble version of IL-6R (sIL-6R) and membrane-bound gp130. Trans-signaling, but not the classical pathway, is regulated by soluble gp130. In this review, we discuss the similarities and differences in IL-6 cytokine and myokine signaling to explain the differential and opposite effects of this protein during inflammation and exercise, with a special focus on the vascular system.

## Introduction

Interleukin-6 (IL-6) is the principal member of the cytokine IL-6 superfamily (White and Stephens, 2011; Tanaka et al., 2014). This protein is comprised of 212 amino acids and has a mass of 21–26 kDa. As a cytokine, IL-6 participates in the innate immune response (Geiger et al., 1988). IL-6 potently induces acute-phase proteins, C-reactive protein (CRP), several complement system proteins, and the coagulation cascade (Geiger et al., 1988; Sproston and Ashworth, 2018). IL-6 also regulates body thermogenesis by acting as an endogenous pyrogen; stimulates hematopoietic precursor growth; and promotes T and B lymphocyte differentiation and maturation (Mihara et al., 2012; Evans et al., 2015).

IL-6 acts not only as a cytokine, however, but also as a myokine, expressed and released by skeletal muscle during exercise (Raschke and Eckel, 2013). As a myokine, IL-6 acts in a paracrine and autocrine fashion in skeletal muscle and an endocrine hormone-like fashion to mediate anti-inflammatory and metabolic processes (Pedersen, 2013). IL-6 triggers an anti-inflammatory response by inducing expression of anti-inflammatory factors such as IL-1ra (IL-1 receptor agonist) and IL-10 and reducing production of the pro-inflammatory cytokines TNFα and IL-1ß (Eckardt et al., 2014). IL-6 also plays a role in hypertrophic skeletal muscle growth (Serrano et al., 2008). Metabolic effects of IL-6 in humans include improved insulin signaling, enhanced insulin sensitivity, and increased fatty acid oxidation in skeletal muscle (Catoire and Kersten, 2015).

Most literature on this glycoprotein is related to its immunoregulatory and proinflammatory actions. A PubMed search for IL-6 in inflammation and immune response displayed 84,159 articles, while a query on the role of IL-6 as a myokine in exercise produced only 3,905 results. This disparity is also reflected in the abundance of information on IL-6 as an immunoregulatory and pro-pathogenic molecule, with much scarcer data on beneficial IL-6 activity. Despite the wealth of research on IL-6, the exact mechanism that regulates the balance between its detrimental and favorable effects remains elusive. The most-accepted theory to explain this dual behavior involves a complex IL-6-dependent signaling network comprised of the classical and trans-signaling pathways.

## IL-6 Signaling

**FIGURE 1:**
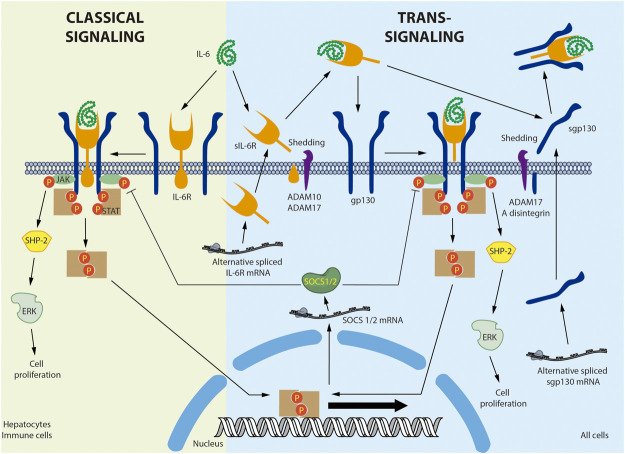
**Interleukin-6 classical and trans-signaling.** In the classical signaling, interleukin-6 (IL-6) binds to a membrane bound-IL-6 receptor (IL-6R) triggering the attachment and dimerization of glycoprotein 130 (gp130), followed by the activation of the non-receptor tyrosine kinase JAK that phosphorylates the STAT transcription factors. The activation of non-canonical signaling pathways, such as SHP-2/ERK, was also described. In the trans-signaling, soluble IL-6R (sIL-6R) was produced by shedding of the membrane bound IL-6R by ADAM10 or ADAM17. In human, but not in mice, sIL-6R was also produced by alternative splicing. IL-6 interacts with sIL-6R and the IL-6/sIL-6R complex binds a membrane bound gp130, activating the same signaling pathway described for classical signaling. Soluble gp130 (sgp130) was produced mainly by alternative splicing and also by shedding of the membrane bound gp130 by ADAM17 and A disintegrin. sgp130 binds the IL-6/sIL-6R complex and selectively inhibits the trans-signaling without affecting the classical signaling. Negative feedback involves the Stat-dependent expression of SOCS1/2 that inhibit JAK activity. Membrane bound IL-6 was described in hepatocytes and immune cells. Membrane bound gp130 is ubiquitous expressed in all cells.

Figure 1 provides a detailed overview of IL-6 signaling. IL-6 binds to the plasma membrane-associated IL-6 receptor (IL-6R). IL-6R is an 80-kDa glycoprotein with a cytoplasmic domain of only 82 amino acids. Given the short span of its intracellular domain, IL-6R is unable to transduce the signal alone (Varghese et al., 2002). The IL-6/IL-6R complex associates with the signal transducer glycoprotein 130 (gp130). In contrast to IL-6R, gp130 has a 277-amino acid cytoplasmic domain containing several phosphorylation sites and scaffolding motifs where the signal can be transduced (Wolf et al., 2014). Binding of IL-6 to its receptor induces gp130 homodimerization and activation (Taga and Kishimoto, 1997). The active complex recruits the JAK non-receptor tyrosine kinase, which phosphorylates the tyrosine residues of gp130 (Babon et al., 2014). gp130 phosphorylation in turn generates recruitment sites for other proteins such as SHP-2/ERK and STAT1/3, activating multiple signaling cascades (Schaeffer et al., 2001). IL-6-dependent ERK activation is associated with cell proliferation (Daeipour et al., 1993; Saad et al., 2019). Moreover, STAT3 activation induces inhibitory molecules such as SOCS1 and SOCS3, which bind to JAK and gp130, respectively, to generate a negative feedback loop (Narazaki et al., 1998; Nicholson et al., 2000). This mechanism is known as the classical IL-6 signaling pathway (Demyanets et al., 2012).

A soluble IL-6R (sIL-6R) has been described in various body fluids, including blood (Wolf et al., 2016). sIL-6R is preferentially produced by membrane IL-6R shedding by metalloprotease A disintegrin and metalloproteinase 17 (ADAM17) (Mullberg et al., 1993; Riethmueller et al., 2017). sIL-6R is also produced by an alternatively spliced mRNA in humans, but not mice (Lust et al., 1992). In circulation, IL-6 can bind to the soluble receptor and exist as an IL-6/sIL-6R complex, increasing the half-life of IL-6 (Rosa Neto et al., 2009). gp130 is expressed by all cells in the body, whereas membrane-bound IL-6R is expressed primarily by hepatocytes and various inflammatory cells, mainly neutrophils, monocytes, macrophages, granulocytes, Kupffer cells, eosinophils, T regulatory cells (Treg), memory CD4^+^ T cells, naïve T cells, dendritic cells, basophils, naïve CD8^+^ cells, and memory CD8^+^ cells (Taga and Kishimoto, 1997; Schmiedel et al., 2018; Monaco et al., 2019). Therefore, unlike IL-6, the soluble sIL-6R/IL-6 complex can bind to and stimulate cells that only express gp130. This last mechanism is referred to as trans-signaling (Wolf et al., 2016). It has been broadly accepted that classical IL-6 signaling is associated chiefly with the immune response and trans-signaling with more systemic processes. However, this assumption may need to be reconsidered, as single-cell analysis of several tissues, including the heart (Wang et al., 2020), lung (Vieira Braga et al., 2019), and kidney (Liao et al., 2020), has identified IL-6R in cardiomyocytes, vascular smooth muscle cells, fibroblasts, type 1 and 2 alveolar cells, ciliated cells, endothelial cells, and proximal tubular cells. Nonetheless, proteomic analysis of these cells must be completed before conclusions can be drawn regarding the role of the classical pathway in these tissues.

gp130 also exists in a soluble form. Although soluble gp130 (sgp130) is preferentially produced by alternative splicing, it can also be generated through shedding by ADAM10 and ADAM17 (Wolf et al., 2016). sgp130 interacts with the IL-6/sIL-6R complex but not with IL-6 alone (Jostock et al., 2001). Therefore, the function of sgp130 is to selectively capture the IL-6/sIL-6R complex, thus inhibiting IL-6 trans-signaling without disrupting classical IL-6 signaling (Demyanets et al., 2012; Wolf et al., 2016). In fact, specific trans-signaling inhibition with sgp130 has been shown to have beneficial effects in inflammatory diseases and atherosclerosis in animal models (Morieri et al., 2017). Therefore, it has been proposed that the anti-inflammatory and regenerative activities of IL-6 are mediated by classical signaling, while its pro-inflammatory actions are mediated by trans-signaling (Scheller et al., 2011). However, this assertion is questionable, as the IL-6/sIL-6R complex stimulates glucose transport in skeletal muscle, increasing AMPK phosphorylation (Gray et al., 2009b). Moreover, trans-signaling in the central nervous system suppresses feeding and improves glycemic control, effects that seem to be enhanced in obese mice (Timper et al., 2017). Moreover, because both classical and trans-signaling activate the same transduction cascades downstream of gp130 ((Mihara et al., 2012; Tanaka et al., 2014; Rose-John, 2018), factors differentially involved in both pathways, such as IL-6R, and sgp130, the kinetics and tissue-specific expression should be also considered in this discussion.

## IL-6 Regulation of Innate and Adaptative Responses

IL-6 controls both innate and adaptative immune responses. In fact, IL-6-knockout mice show impaired innate and adaptive immunity to infection by parasites, bacteria, and viruses (Kopf et al., 1994). IL-6 regulation of the innate immune response involves several elements. Activation of pattern recognition receptors, such as Toll-like receptors, induces IL-6 secretion by neutrophils and monocytes or macrophages (Chalaris et al., 2007). Moreover, stromal cells—including fibroblasts, myofibroblasts, endothelial cells, smooth muscle cells, and epithelial and mesothelial cells—also secrete IL-6 upon pattern recognition receptor activation (West, 2019). Furthermore, activation of the complement system, in particular the C5a receptor, induces the release of IL-6 in human osteoblast-like cells (Pobanz et al., 2000) and enhances the release of IL-6 in neutrophils exposed to lipopolysaccharide (LPS) (Riedemann et al., 2004). These data suggest a complex interplay between IL-6 and innate immune response activation. On the other hand, IL-6 also regulates innate immunity by controlling innate immune cell activity (Jones and Jenkins, 2018). IL-6 triggers the recruitment, adhesion, activation, differentiation, and survival of neutrophils, tissue-resident and inflammatory monocytes, and innate lymphoid cell populations including natural killer cells (Rose-John et al., 2017; Jones and Jenkins, 2018). Additionally, IL-6R is shed by neutrophils (Chalaris et al., 2007) and activates trans-signaling of stromal cells. Activated stromal cells secrete various chemokines through a NF-κB-dependent mechanism, attracting monocytes and/or macrophages to resolve inflammation (Hurst et al., 2001). In this context, the initial neutrophil infiltration activates both classical and trans-signaling to amplify and modulate the innate immune response at the infection site.

Adaptive immune response regulation by IL-6 depends on its ability to control T helper cell differentiation (Bettelli et al., 2006). Type 1 T helper (Th1), type 2 T helper (Th2), type 17 T helper (Th17), and type 22 T helper (Th22) cells are well-recognized activators of the immune response, while regulatory T (Treg) cells are known to inhibit T-cell activation (Chatzileontiadou et al., 2020). Th17 cells, discovered in 2005, produce IL-17A, IL-17F, IL-22, and TNFα (Harrington et al., 2005). In mice, incubation of naïve T cells with transforming growth factor (TGF)-β induces the Treg differentiation that produces IL-10. These cells therefore have significant anti-inflammatory and regulatory properties. In the presence of IL-6, on the other hand, TGF-β promotes Th17 cell differentiation (Bettelli et al., 2008; Korn et al., 2008), and IL-6-knockout mice cannot generate Th17 cells (Korn et al., 2008). Moreover, IL-6R shedding upon T-cell receptor activation has been described (Briso et al., 2008). Consequently, it can be hypothesized that after initial activation of classical IL-6 signaling, IL-6 trans-signaling is required to effectively stimulate Th17 differentiation (Dominitzki et al., 2007). IL-6 is also involved in B-cell growth, plasma cell differentiation (Suematsu et al., 1989), and class switching (Dienz et al., 2009). Finally, IL-6-deficient mice show diminished antigen-induced increases in IgG1, IgG2a, and IgG3, but not IgM (Kopf et al., 1998).

Apart from its proinflammatory actions, IL-6 can inhibit lipopolysaccharide (LPS)-induced TNF-α in cultured human monocytes (Schindler et al., 1990). Both recombinant IL-6 infusion and exercise inhibit the LPS-induced increase in TNFα in healthy individuals (Starkie et al., 2003). In a model of concanavalin A-induced T cell activation-associated hepatic injury, recombinant IL-6 induces a protective effect by reducing TNFα production (Mizuhara et al., 1994). Furthermore, in young healthy individuals, a single dose of IL-6 stimulates IL-1ra and IL-10 (Steensberg et al., 2003). Because the IL-6 signaling pathway components were not fully described in these experiments, it is not possible to determine whether these anti-inflammatory actions are mediated by classical or trans-signaling. More work is required to fully elucidate the mechanism involved in the switching of pro-inflammatory to anti-inflammatory actions of IL-6.

## Exercise and Vascular Remodeling

Cardiovascular diseases (CVD) are mainly triggered by other vascular diseases, i.e., coronary and cerebrovascular diseases. CVDs are the leading cause of mortality and morbidity worldwide (Mensah et al., 2019). Moderate-intensity exercise is considered essential for maintaining cardiovascular health (Morris et al., 1953). The beneficial effects of moderate exercise on traditional risk factors (obesity, hypertension, diabetes, and hypercholesterolemia) may explain approximately half of the risk reduction associated with exercise (Joyner and Green, 2009). It has been proposed that direct effects of moderate exercise on the vessels may account for some of the remaining “risk factor gap” (Joyner and Green, 2009). Training can stimulate both formation of new capillaries by angiogenesis and increased conduit artery size by arteriogenesis (Green and Smith, 2018). Structural adaptations in the vessels, induced by repeated exercise bouts, involves arterial enlargement without fibrosis or immune cell infiltration (Green and Smith, 2018). These data support the idea that exercise induces physiological vascular remodeling. These changes increase blood flow in skeletal muscles and other organs to fulfill the nutrient and oxygen requirements of athletes. This vascular remodeling is known as the “athlete’s artery” (Green et al., 2012). Various mediators of exercise-induced vascular remodeling have been proposed, including vascular endothelial growth factor (VEGF), angiopoietins 1 and 2, fibroblast growth factor 2 (FGF2), and others (Prior et al., 2003). We suggest that specific myokines, particularly IL-6, may also participate in exercise-induced vascular remodeling.

## IL-6 and Exercise

Plasma IL-6 levels range from 1 to 10 pg/ml in healthy individuals. sIL-6R and sgp130, on the other hand, are present at much higher levels in the plasma, at 25–75 ng/ml and 100–400 ng/ml, respectively (Montero-Julian, 2001; Baran et al., 2018). IL-6 content in skeletal muscle is low at rest, with small amounts of IL-6 found mainly in type I fibers (Plomgaard et al., 2005). Basal IL-6 levels appear to be regulated by training. Epidemiological studies have found negative associations between volume of regular physical activity and basal plasma IL-6 levels (Pitsavos et al., 2005). Basal IL-6 was also reduced in obese postmenopausal women subjected to regular aerobic exercise (225 min/week of moderate-to-vigorous activity) and a hypocaloric diet (Imayama et al., 2012). Coronary artery disease patients and adults >64 years old showed similar training-induced effects (Goldhammer et al., 2005; Kohut et al., 2006). However, other reports have found no effect of training on basal IL-6 levels (Leggate et al., 2012; Isaksen et al., 2019).

Nearly all studies conducted to date have demonstrated increased plasma IL-6 levels in response to various types of acute exercise (Catoire and Kersten, 2015). A correlation between increased plasma and muscle IL-6 mRNA has also observed (Catoire and Kersten, 2015). These increases occur exponentially, and the peak, usually about 100-fold over basal levels, is reached immediately at the end of the exercise session and quickly returns to pre-exercise levels (Croisier et al., 1999; Sabaratnam et al., 2018). The magnitude of the increase in IL-6 levels is related to the type, duration, and intensity of the exercise, as well as the amount of muscle mass engaged (Pedersen, 2013; Catoire and Kersten, 2015). Increased IL-6 mRNA expression is normally observed after 30 min of exercise (Steensberg et al., 2002). However, elevations in acute IL-6 mRNA expression have also been observed in skeletal muscle during high-intensity training (Eaton et al., 2018).

IL-6 expression is more sensitive to exercise duration than intensity. In fact, approximately 51% of the variation in IL-6 plasma levels depends on exercise duration (Fischer, 2006; Robson-Ansley et al., 2009). For some types of exercise such as running, bicycling, or eccentric training, the most pronounced increases in plasma IL-6 are observed in association with intense weight-bearing or endurance drills, which involve several large muscle groups and deplete glycogen storage (Fischer, 2006; Catoire and Kersten, 2015). Few studies have evaluated the effect of training on exercise-induced elevations in plasma IL-6. One study assessed the effects of a 10-weeks program consisting of 1 h of knee-extension exercises 5 times per week. A group of seven healthy men showed less marked elevations in post-exercise skeletal muscle IL-6 mRNA after training, but no change in post-exercise plasma IL-6 (Fischer et al., 2004). Another study showed that resistance training reduces IL-6 mRNA in skeletal muscle, likely by establishing an adaptation mechanism that prevents abrupt changes in IL-6 concentration (Gokhale et al., 2007).

In short, exercise induces transitory increases in IL-6, while inflammation induces more sustained elevations (Fischer, 2006; Munoz-Canoves et al., 2013). It has been proposed that this distinction could explain the dual effects of IL-6. Studies involving athletes who perform chronic, very frequent, strenuous training, such as highly trained professional athletes, might clarify whether this kind of exercise could produce a more chronic increase in plasma IL-6 levels and whether or not such a change might have detrimental effects.

## sIL-6R and Exercise

Physical exercise, in addition to raising plasma IL-6 concentration, can increase levels of sIL-6R and therefore the IL-6/sIL-6R complex (Gray et al., 2009a). Training also modifies basal plasma sIL-6R levels. A 12-weeks physical training program reduced basal plasma sIL-6R in 24 patients with stable congestive heart failure (Adamopoulos et al., 2002). Moreover, a hypocaloric diet and exercise reduced basal plasma sIL-6R in 17 obese postmenopausal women (You et al., 2004). A comparable decrease was observed in 12 obese males subjected to 2 weeks of high-intensity intermittent training (Leggate et al., 2012). After prolonged exercise, IL-6 levels increase transiently, while sIL-6R increases persistently, which may partly explain the fatigue at rest that occurs after physical activity (Robson-Ansley et al., 2009). Therefore, increased IL-6 trans-signaling would be expected during exercise. However, a group of 12 healthy subjects subjected to a submaximal bout of cycling to volitional exhaustion also showed increased plasma sgp130 levels (Gray et al., 2008). This effect was not observed with less-intense exercise (Patterson et al., 2008). These data suggest that both classical and trans-signaling are activated and regulated during strenuous exercise by complex interactions among IL-6, sIL-6R, and sgp130. However, whether the kinetics of induction and balance of these molecules are responsible for the beneficial effects of IL-6 induced by exercise remains unknown.

## IL-6 in Adipose Tissue

IL-6 is one of the most-studied myokines associated with communication between skeletal muscle and white adipose tissue (Fischer, 2006; Mihara et al., 2012; Raschke and Eckel, 2013; Ahima and Park, 2015; Timper et al., 2017; Rose-John, 2018). *In vitro*, IL-6 induces lipolysis in adipocytes and fatty acid oxidation in myotubes (Carey et al., 2006; Yang et al., 2008). In diabetic patients and controls, acute IL-6 treatment increases fatty acid turnover (Petersen et al., 2005). Moreover, administration of tocilizumab, an IL-6R-blocking antibody, to abdominally obese individuals blocks exercise-induced reductions in visceral adipose tissue mass (Wedell-Neergaard et al., 2019). These data suggest that IL-6 induces fatty mobilization from adipose tissue to the blood, making the fatty acids available to the muscle during exercise.

In obese ob/ob mice, adipocyte-specific deletion of IL-6 induced an approximately 40% reduction in plasma IL-6 levels (Whitham et al., 2019). These data suggest that adipose tissue is a major contributor to basal plasma IL-6 levels in obesity. Accordingly, a significant increase in the release of IL-6 from visceral vs. subcutaneous fat has been observed in obese individuals (Jonas et al., 2015). Because adipocytes express very low levels of IL-6R (Path et al., 2001), IL-6 trans-signaling is considered the main mechanism of IL-6 action. Inhibition of IL-6 trans-signaling by sgp130 prevents high-fat diet-induced adipose tissue macrophage recruitment but does not improve insulin resistance (Kraakman et al., 2015). Furthermore, an adipocyte-specific gp130-knockout mouse model was used to demonstrate that adipocyte-specific IL-6 trans-signaling is involved in exercise-mediated regulation of food intake and weight reduction in mice fed a high-fat diet (Odermatt et al., 2020). This action could be explained by the suppression of feeding triggered by IL-6 through the regulation of the expression of neuropeptides at the hypothalamic arcuate nucleus (Senaris et al., 2011; Schele et al., 2013).

On the other hand, browning of white adipose tissue is a promising strategy for treating obesity (Lizcano and Arroyave, 2020). An increased mitochondrial uncoupling protein 1 (UCP1) level is considered a marker of adipose cell browning (Lizcano and Arroyave, 2020). Whole-body deletion of IL-6 completely prevents the increase in uncoupled protein-1 (UCP-1) mRNA and protein induced by both cold exposure and exercise (Knudsen et al., 2014). However, it remains unknown whether classical or trans-signaling is involved in IL-6-induced adipocyte browning.

## IL-6 and Arteries

**FIGURE 2:**
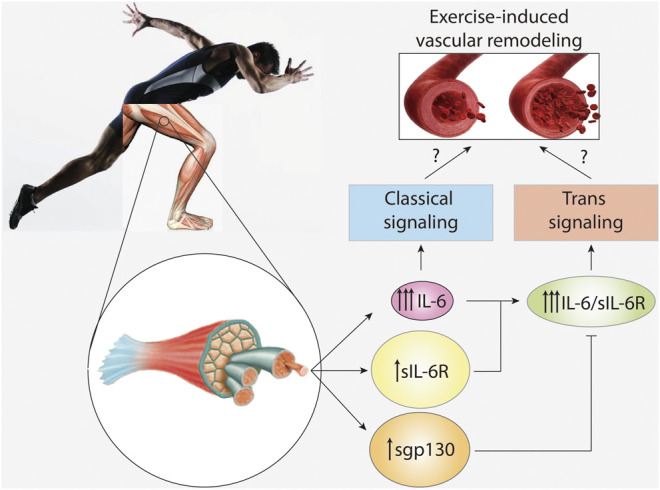
**IL-6 signaling in vascular remodeling induced by exercise.** High intensity and long duration exercises induce a large plasma level increase of interleukin-6 (IL-6), and a modest increase in soluble IL-6 receptor (sIL-6R) and soluble glycoprotein 130 (sgp130). However, basal levels of sIL-6R and spg130 are at least hundred times higher than those of IL-6. IL-6 activates classical signaling, whereas the complex IL-6/sIL-6R activates trans-signaling. Trans-signaling is specifically regulated by sgp130. The role of IL-6 signaling and trans-signaling in vascular remodeling induced by exercise is unknown.

Vascular smooth muscle cells (VSMC) are the main component of the medial layer of the artery. These cells contract to regulate blood vessel tone and thus blood flow and pressure (Chiong et al., 2013). VSMC also have the capacity to secrete molecules, allowing for synthesis and repair of extracellular matrix proteins and regulation of vascular wall structure (Cecchettini et al., 2011). Normal VSMC are non-fully differentiated cells with very low rates of proliferation and secretion (Cecchettini et al., 2011). Exercise-induced increases in arterial diameter due to arteriogenesis are characterized by a phenotypic switch in VSMC from a contractile to a migratory and proliferative state (Cui et al., 2009; Poling et al., 2011). Chronic exposure to flow changes and shear stress leads to carotid artery wall thinning and increased VSMC proliferation (Figure 2) (Green et al., 2017). In addition, the release of growth factors such as PDGF, which induces VSMC migration and proliferation, has also been detected during exercise (Green et al., 2017).

IL-6 is produced by a variety of cells in addition to the skeletal muscle, including monocytes (Gauldie et al., 1987), epithelial cells (Von Patay et al., 1998), cardiomyocytes (Millan et al., 1987), and VSMC (Loppnow and Libby, 1990; Viedt et al., 2002). In VSMC, IL-6 expression is induced by pro-inflammatory stimuli such as IL-1 (Loppnow and Libby, 1990), the monocyte chemoattractant protein-1 (MCP-1) (Viedt et al., 2002), oncostatin M (Schnittker et al., 2013), lipopolysaccharides (Schnittker et al., 2013), CRP (Hattori et al., 2003), and TNF-α (Garcia-Miguel et al., 2018). Non-inflammatory stimuli such as PDGF may also induce IL-6 expression in VSMC (Roth et al., 1995). Acute exercise increases IL-6 mRNA levels in adipose tissue (Holmes et al., 2004), suggesting that exercise-dependent plasma IL-6 might also originate from tissues other than skeletal muscle (Catoire and Kersten, 2015). However, it remains to be seen whether exercise also induces IL-6 in VSMC.

Human VSMC constitutively express small amounts of both membrane-bound IL-6R and gp130 (Klouche et al., 1999). However, stimulation with the IL-6/sIL-6R complex provoked a marked upregulation of gp130, suggesting induction of IL-6 trans-signaling (Klouche et al., 1999). Moreover, treating cultured VSMC with IL-6 alone also induces cell proliferation and migration (Morimoto et al., 1991; Wang and Newman, 2003), reduces VSMC contractility (Ohkawa et al., 1994), and induces matrix metalloproteinase (MMP)-9 and MMP-1 production (Zhu et al., 2000). These data suggest that both classical and trans-signaling mechanisms are at play in VSMC (Klouche et al., 1999). However, no study to date has clarified whether IL-6 and/or IL-6/sIL-6R induce exercise-dependent artery remodeling *in vivo* (Figure 2).

## IL-6 in Vascular Diseases

Pathological changes in the VSMC phenotype have been widely described in the development and progression of neointimal formation, hypertension, and atherosclerosis (Campbell and Campbell, 1985; Cecchettini et al., 2011). Pathological vascular remodeling is characterized by narrowing of the vessel lumen, mobilization of muscle cells to the intima, exacerbation of extracellular matrix production (fibrosis), and infiltration by immune cells (Renna et al., 2013). In addition to increased proliferation and migration rates, VSMC phenotypic switching involves increased extracellular matrix component production, altered expression of contractile proteins, and production of proteases and pro-inflammatory cytokines (Campbell and Campbell, 1985). Processes such as proliferation, contraction, secretion, and migration in VSMC are affected by a wide range of factors, including mechanical forces, reactive oxygen species, extracellular matrix components, contractile agonists such as angiotensin II, endothelial-VSMC interactions, transforming growth factor (TGF)-β1, PDGF, and many other growth factors (Campbell and Campbell, 1985; Cecchettini et al., 2011).

Plasma IL-6 levels are used as a marker for CVD such as coronary artery disease and atherosclerosis (Kinlay and Egido, 2006). Elevated IL-6 expression has also been detected in atherosclerotic lesions (Schieffer et al., 2000). Administration of IL-6 to male mice fed normal or high-fat diets exacerbated atherosclerosis (Huber et al., 1999). Moreover, treating ApoE-deficient mice with an IL-6-reducing agent (Am80) resulted in smaller lesions as compared to untreated mice (Takeda et al., 2006). These results may be associated with the pathological effects of IL-6. However, opposite effects were observed in atherosclerosis-prone C57BL/6 and ApoE-deficient mice. Increasing IL-6 levels reduced atherosclerotic lesion size in both animal models (Loppnow et al., 2011). Consistent with these results, Schieffer et al. observed reduced monocyte recruitment and increased lesion size in ApoE- and IL-6-deficient as compared to wild-type mice (Schieffer et al., 2004). Ovariectomized female ApoE/IL-6-knockout mice fed a normal diet for one year also developed larger lesions than IL-6-expressing wild-type mice (Elhage et al., 2001). The last three results suggest that IL-6 protects against, rather than promoting, atherosclerotic lesion formation. Therefore, more studies dissecting the IL-6-induced classical and trans-signaling is required to clarify the beneficial and detrimental effects of this protein in the vascular bed.

Pulmonary artery hypertension (PAH) involves various medical conditions in which the pulmonary circulation blood pressure is significantly increased (Mclaughlin et al., 2009). Mechanisms involved in the genesis of PAH include hypoxia-induced pulmonary artery smooth muscle cell (PASMC) proliferation and cell death resistance, leading to pathological narrowing of the pulmonary circulation and a consequent increase in pulmonary artery blood pressure (Parra et al., 2017). Increased numbers of macrophages are present within the pulmonary lesions of patients with severe PAH (Gerasimovskaya et al., 2012). In mice, hypoxia induces macrophage activation, triggering the proinflammatory milieu characteristic of PAH (Vergadi et al., 2011). IL-6 is proposed as one of the primary cytokines involved in the pathogenesis of PAH and hypoxia-induced pulmonary hypertension (Groth et al., 2014). Moreover, mice with lung-specific IL-6-overexpression show increased pulmonary vascular remodeling, characterized by increased muscularization of the proximal arterial tree (Steiner et al., 2009). This condition is due to the induction of PAMSC proliferation and inhibition of apoptosis (Steiner et al., 2009). This effect of IL-6 is similar to those described for non-pulmonary arteries described above. Conversely, in whole-body IL-6-deficient mice, hypoxia fails to induce PAH (Savale et al., 2009). Because serum levels of IL-6 and sIL-6R, but not spg130, are increased in PAH patients, it is thought that IL-6 trans-signaling may be involved in PAH pathogenesis (Jasiewicz et al., 2015). However, this relationship has yet to be demonstrated.

IL-6 has also been implicated as a mediator of other respiratory diseases, including pneumonia, acute respiratory distress syndrome, and Covid-19 (Chalmers et al., 2019; Herold et al., 2020; Liu et al., 2020). In these patients, acute pulmonary injury is associated with a hyper-inflammatory state that predicts a worse clinical outcome (Spadaro et al., 2019; Herold et al., 2020; Liu et al., 2020). However, inhibiting IL-6 signaling to limit cytokine-dependent lung injury, using sarilumab or tocilizumab for example, has only been explored in Covid-19 patients (Rossotti et al., 2020; Sinha et al., 2020), rheumatoid arthritis-related interstitial lung disease (Manfredi et al., 2020; Vacchi et al., 2020), and PAH (Hernandez-Sanchez et al., 2018). More systematic studies clarifying the inflammatory and vascular remodeling effects of IL-6 in the lung is required to fully understand the role of this cytokine in pulmonary diseases.

## Projections

Repetitive strenuous exercise induces artery remodeling to fulfill the nutrient and oxygen demands of the skeletal muscles. This vascular remodeling is likely elicited by myokines. Although IL-6 is the myokine that has received the most research attention, the complex nature of IL-6 regulation, which involves interplay among several molecules including IL-6, IL-6R, gp130, sIL-6R, and sgp130, makes it challenging to dissect the cytokine and myokine actions of IL-6. Moreover, plasma levels of these molecules are tightly regulated depending on exercise type, duration, and intensity. Because the secretion of IL-6 is also triggered by glucagon-like peptide 1, an incretin released by food intake (Shirazi et al., 2013), and IL-6 induces suppression of food intake and reduction of body weight, an interesting cross-talk between exercise and food consumption could exist through the regulation of IL-6 levels. New strategies involving IL-6 regulation by food intake should be interesting to explore. Although IL-6 induces VSMC dedifferentiation *in vitro*, more detailed and controlled experiments are required to clarify the suspected role of IL-6 in exercise-induced vascular remodeling. Such studies may unravel the mechanisms involved in the beneficial effects of exercise-induced IL-6, and the findings could be used to intervene in both the pro-inflammatory and detrimental actions of IL-6, leading to new treatments for IL-6 dependent chronic inflammatory diseases.
